# Genome-Wide Identification and Expression Analysis of the NF-YB Gene Family in Wheat (*Triticum aestivum* L.) Under Short-Term Salt Stress

**DOI:** 10.3390/ijms272110009

**Published:** 2026-11-08

**Authors:** Wei Gao, Ruixin Zan, Yulu Li, Xinyu Liu, Li Liu, Chong Xue, Yang Zhang, Huiyu Zhang, Xiaoli Li

**Affiliations:** 1 Wheat Research Institute, Shanxi Agricultural University, Linfen 041000, China; ninan0861@163.com (W.G.); m15936507900@163.com (R.Z.); 15064781671@163.com (Y.L.); 15239168947@163.com (X.L.); liuli2883@126.com (L.L.); 17535705813@163.com (C.X.); yangzhang610@163.com (Y.Z.); 2 Agricultural College, Shanxi Agricultural University, Jinzhong 030810, China

**Keywords:** wheat, NF-YB gene family, expression analysis, salt stress, bioinformatics analysis

## Abstract

The transcription factor NF-YB (nuclear factor-YB) family is a subfamily of the nuclear factor Y (NF-Y) family, which regulates plant development and abiotic stress responses. Though extensively studied in many plants, systematic indepth analyses of NF-YB genes in wheat remain scarce. To clarify the characteristics of the wheat NF-YB gene family, this study used the latest China Spring wheat genomic data and bioinformatics methods to analyze this gene family and its encoded proteins. The results showed that 34 *TaNF-YBs* are unevenly distributed across wheat chromosomes, and all encoded proteins are predicted to localize in the nucleus. Analysis of conserved domains, phylogenetic relationships, and gene structure confirmed the high conservation of *TaNF-YBs*. Computational gene collinearity analysis indicated that segmental duplication is the main mechanism underlying the expansion of the TaNF-YB gene family. This study also indicated that the predicted cis-regulatory elements in the promoter regions of *TaNF-YBs* putatively exhibit responsiveness to light, methyl jasmonate (MeJA) and abscisic acid (ABA) responsiveness. Tissue expression profiles revealed that *TaNF-YBs* exhibit high expression in seeds, which may be associated with grain development and nutrient accumulation. The temporal expression profiles of these family genes under salt stress differed significantly. GO enrichment and in silico protein–protein interaction analyses further suggest that TaNF-YB proteins may potentially activate the transcriptional expression downstream salt-tolerance target genes through heterotrimeric complex binding to their promoter regions, thereby enhancing wheat salt-stress responses. This study demonstrates that the TaNF-YB gene family in wheat may have a dual role in both grain development and the response to salt stress, providing theoretical and genetic resources for breeding stress-resistant high-quality wheat.

## 1. Introduction

Nuclear factor-Y (NF-Y) transcription factors (TFs) are widely distributed conserved heterotrimeric complexes in eukaryotes, composed of three subunits: NF-YA (CBF-B/HAP2), NF-YB (CBF-A/HAP3), and NF-YC (CBF-C/HAP5), sometimes referred to as heme-activated proteins [1,2]. These subunits exhibit distinct structural configurations. The NF-YA subunit is localized in the nucleus and primarily binds to the CCAAT box within target gene promoters; it contains two α-helix domains (A1 and A2): the A1 domain, located in the N-terminal core region, mediates binding with both NF-YB and NF-YC subunits and consists of 20 amino acids; the C-terminal A2 domain harbors the specific sequence required for CCAAT binding and recognition [2,3,4,5,6]. Both NF-YB and NF-YC subunits possess a histone folding domain/motif (HFD/HFM) composed of three α-helices (α1, α2, and α3), which is essential for protein–DNA and protein–protein interactions [7,8]. The α1 helix in NF-YB contains a proposed DNA-binding domain, whereas the α1 helix in NF-YC is composed of conserved amino acids that may function as a DNA-binding domain [5]. Since all NF-YB subunits lack nuclear localization signals (NLSs), they must form heterodimers with NF-YC subunits [9,10]. For example, *bZIP67* interacts with *NF-YB9-NF-YC2* to directly bind ABA-responsive elements in the promoter regions of SUS2 and CRC (sucrose synthase 2 and crucifer protein C), thereby activating the expression of Arabidopsis seed development-related genes [11]. In most complex formation and gene expression regulation processes, NF-Y serves as the primary binding subunit. Consequently, the NF-Y complex collaborates with various factors to modulate different genes, thereby influencing multiple regulatory pathways. NF-Y exhibits diverse biological functions spanning developmental pathways to environmental and pathogen stress responses. *NF-YB9* (LEAFY COTYLEDON 1, *LEC1*) is the first identified NF-Y subunit involved in Arabidopsis embryogenesis and post-embryonic development [11,12].

To date, *NF-YBs* have been identified in various species including *Arabidopsis thaliana* [13,14], rice (*Oryza sativa*) [15], maize (*Zea mays* L.) [16], *Populus trichocarpa* [17], and tomato (*Solanum lycopersicum*) [18]. Arabidopsis harbors 13 annotated *NF-YBs* [14], while rice contains 11 *NF-YBs* [19]. Additionally, NF-YB genes have been identified in rapeseed (14), soybean (32), tung oil tree (7), grape (18), and tomato (29) respectively [20,21,22,23], indicating significant expansion of the NF-YB gene family across plant species.

In Arabidopsis, *AtNF-YB2* promotes early flowering and root growth, while *AtNF-YB3* facilitates flowering in response to high-temperature stress [24,25,26]. Overexpression of *AtLEC1* (*AtNF-YB9*) induces the formation of embryoid structures on leaves [27], and *AtLEC1* has been reported as a critical regulatory factor in zygotic embryo development, seed maturation, and fatty acid synthesis [28,29]. In Arabidopsis, overexpression of soybean *GmNF-YB1* enhances tolerance to salt and drought stress, manifested by increased seed-germination rates, longer root lengths, and reduced water loss under stress conditions [30]. Beyond Arabidopsis, functional studies of NF-YBs have been conducted in several major crops, revealing diverse biological roles. In maize, transgenic lines with overexpressed *ZmNF-YB2* or *ZmNF-YB16* exhibit higher water potential and photosynthetic rates under water-deficient conditions, leading to improved yield [24,31]. Research further demonstrates that *BnLEC1* and *ZmLEC1* increase lipid content in seeds [32,33].

Wheat (*Triticum aestivum* L.) is one of the most widely cultivated food crops globally [34], providing 20% of human caloric and protein intake [35]. Enhancing wheat yield and quality is crucial for ensuring global food security [36]. Grain filling represents the final stage of cereal development and serves as a key rate-limiting factor determining yield [37]. During this phase, substantial amounts of starch and storage proteins accumulate in the endosperm, directly influencing wheat yield and quality [38]. However, previous studies on related gene discovery were limited by incomplete gene models and incomplete gene repertoire coverage. Stephenson et al. [39] identified only 11 TaNF-YB members via EST data, and missed many partially homologous copies across A, B and D subgenomes due to limited early genomic resources. Qu et al. [40] identified 34 TaNF-YB family members by combining draft genome sequences with cDNA cloning, but four genes could not be accurately anchored to chromosomes and their gene models were incomplete. Lei et al. [41] only identified 10 TaNF-YB members, all mainly distributed on chromosomes 1 and 4. To date, the systematic identification of the NF-YB gene family in wheat remains incomplete, and the functional roles of this gene family in grain development have not been fully clarified.

This study utilized the latest genomic data of China Spring wheat to conduct a whole-genome identification of this family, identifying a total of 34 *TaNF-YBs* in wheat. Through bioinformatics analysis, we predicted the physicochemical properties, gene structure, conserved domains, phylogenetic relationships, chromosomal localization, collinearity characteristics, and cis-regulatory promoter elements of these genes. Additionally, we also characterized the expression profiles of *TaNF-YBs* in various wheat tissues and under short-term time-series salt-stress treatments. Using RNA-seq and quantitative reverse transcription–polymerase chain reaction (qRT-PCR) techniques, we identified the *TaNF-YBs* potentially regulating wheat growth and development under salt stress. Gene Ontology (GO) enrichment and predicted protein–protein interaction analyses suggest that TaNF-YBs may primarily enhance salt tolerance through the ABA pathway mediated by heterotrimeric NF-Y complexes, while certain subtypes may exert independent transcriptional regulatory functions. This study aims to elucidate the TaNF-YB gene family in wheat at the whole-genome level, laying the foundation for further exploration of their evolutionary history and functional mechanisms.

## 2. Results

### 2.1. Genome-Wide Identification and Sequence Characterization of the TaNF-YBs in Wheat

**Figure 1:**
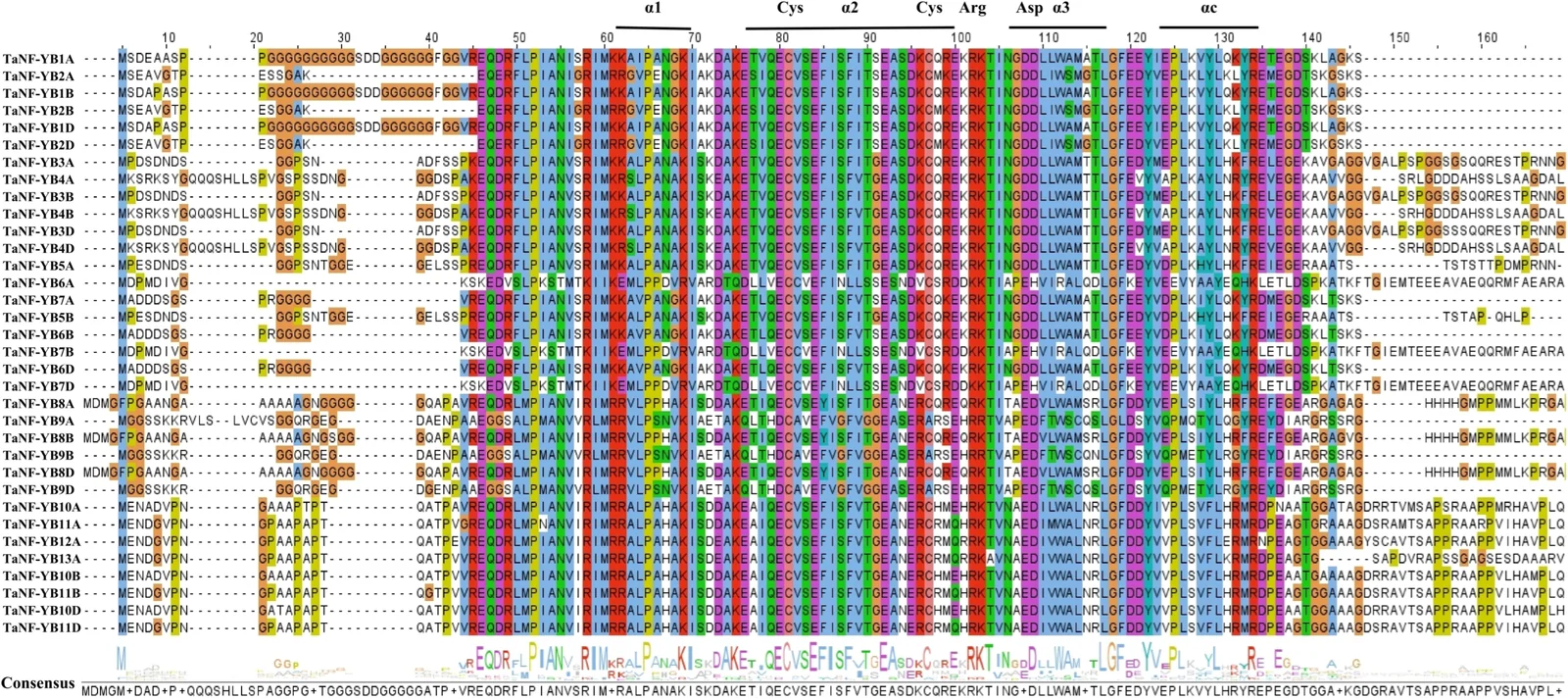
Multiple sequence alignment of TaNF-YB proteins in *Triticum aestivum* L. cv. Chinese Spring. Different colors represent the conservation levels of amino acid residues. The secondary structure elements (α1, α2, α3, αc) and functional annotations of key residues are inferred based on homologous NF-YB structural studies.

Thirty-four members of the NF-YB gene family were identified in the China Spring genome. To elucidate the sequence conservation and structural characteristics of TaNF-YB proteins, multiple sequence alignment analysis was performed on amino acid sequences, yielding conserved sequence motifs and annotations for the NF-YB domain. The results demonstrate that all TaNF-YB proteins contain a highly conserved NF-YB domain (Figure 1 and Figure S1), which comprises four α-helices (α1, α2, α3, and αc). Among these, the α-helices exhibit high conservation across all members, whereas the N-terminal and C-terminal sequences show greater variability. Residue-level conservation analysis indicated that the NF-YB domain has undergone strong purifying selection during evolution. Notably, several functionally critical residues within the NF-YB domain are universally conserved across all TaNF-YBs, including cysteine (Cys) residues that mediate redox-regulated heterodimerization, and a conserved arginine–asparagine (Arg-Asp) pair that maintains the structural integrity of the histone fold motif (HFM). These conserved residues form the structural basis for NF-YB proteins to interact with NF-YA/NF-YC subunits and mediate sequence-specific DNA binding. The high conservation of the core domain suggests that TaNF-YBs likely play essential and conserved roles in wheat growth, development, and stress response mechanisms.

Analysis of the wheat genome annotation files revealed that the 34 *TaNF-YBs* are unevenly distributed across 15 chromosomes (Table 1). Based on their chromosomal locations and homology relationships, these NF-YB genes were systematically designated as *TaNF-YB1A-TaNF-YB13A*. The lengths of their coding sequences (CDS) vary significantly, ranging from 411 bp (*TaNF-YB2A/2B/2D*) to 903 bp (*TaNF-YB10B*), while their amino acid lengths also differ markedly—from 136 aa (*TaNF-YB2A/2B/2D*) to 300 aa (*TaNF-YB10B*)—resulting in substantial differences in predicted protein molecular weights, ranging from 14.89 kDa (*TaNF-YB2B*) to 32.16 kDa (*TaNF-YB7D*). The isoelectric points (pI) of these proteins range from 4.88 (*TaNF-YB13A*) to 6.49 (*TaNF-YB4D*). Plant-mPLoc predicted the subcellular localization of all 34 *TaNF-YBs*, the prediction results suggested that these proteins are likely localized in the nucleus.

### 2.2. Phylogenetic Analysis, Chromosomal Localization, Gene Structure, and Conserved Motifs of TaNF-YBs

**Figure 2:**
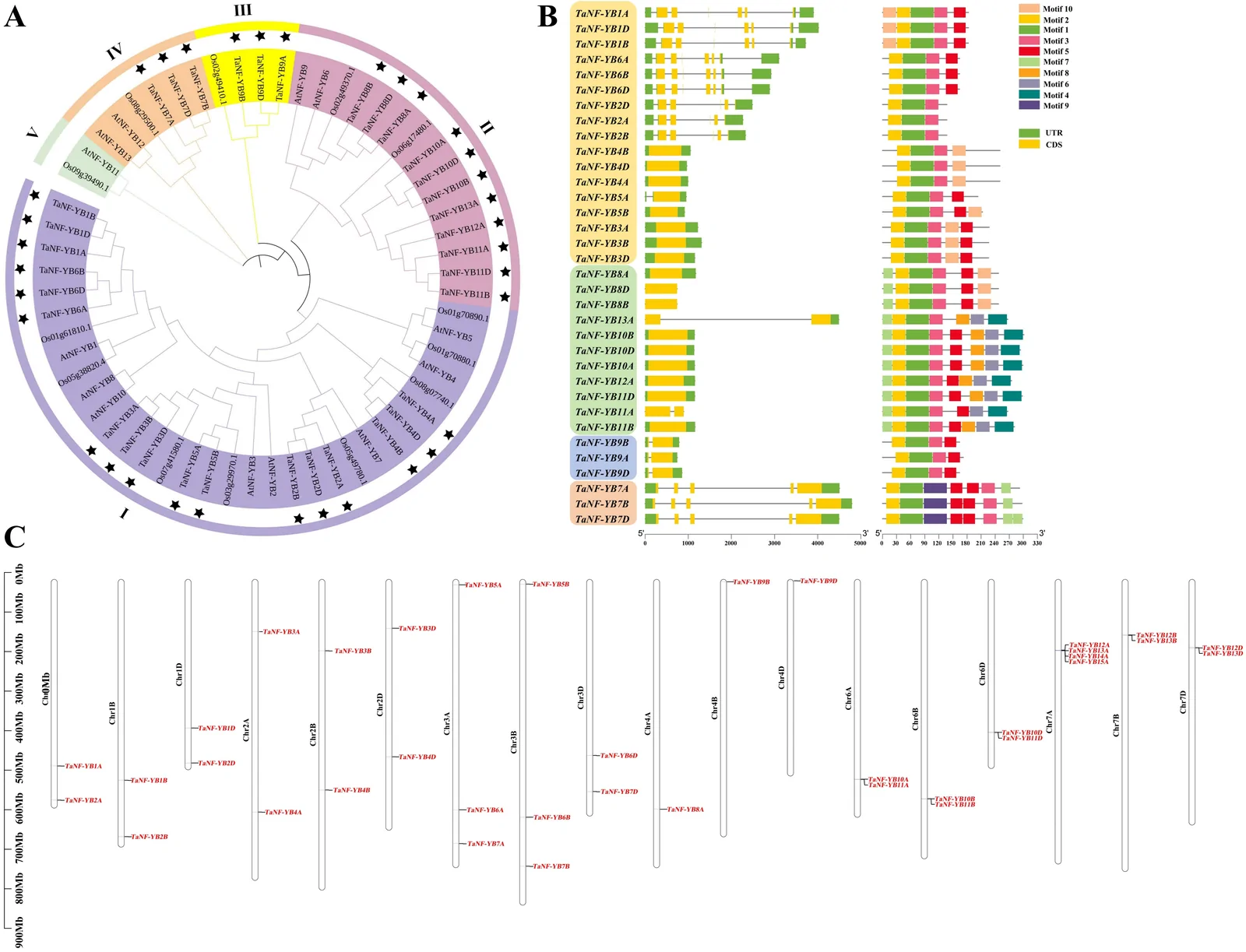
Phylogenetic analysis, gene structure and conserved motifs, and chromosomal distribution of TaNF-YB gene family. (**A**) Phylogenetic analysis of NF-YB in *Triticum aestivum*, *Oryza sativa*, and *Arabidopsis thaliana*. Phylogenetic tree via the NJ method with bootstrap value set to 1000. Light purple, magenta, yellow, orange, and pale green represent subgroups I, II, III, IV, and V respectively. The five-pointed star denotes TaNF-YBs. (**B**) Analysis of the gene structure and conserved motifs of *TaNF-YBs*. Exons are indicated by green boxes, UTRs by yellow boxes, and the black lines connecting two exons represent introns. Ten conserved motifs were computationally identified in TaNF-YB proteins. (**C**) Distribution of *TaNF-YBs* on chromosomes.

To investigate the evolutionary relationships between wheat and other species, phylogenetic trees were constructed for wheat, Arabidopsis, and rice. The results (Figure 2A, Table S1) demonstrate that the NF-YB family can be classified into five subfamilies: Subfamily V lacks TaNF-YBs, while the other four subfamilies contain genes from wheat, Arabidopsis, and rice. Members within each subfamily exhibit close genetic proximity, whereas significant evolutionary divergence is observed between different subfamilies. TaNF-YB can be further divided into four subfamilies, with Subfamily I containing the highest number of members (17), followed by Subfamily II (11), Subfamily III, and Subfamily IV (3 members each). This suggests that the wheat *NF-YB* gene family has undergone evolutionary adaptation, playing distinct regulatory roles in growth development and stress resistance.

To analyze the gene structure and conserved motifs of TaNF-YB proteins, we employed MEME software for structural characterization and TBtools-II v2.322 for visualization. Analysis of 34 *TaNF-YBs* revealed (Figure 2B) that all subfamilies share similar exon-intron structures, with greater structural similarity among members within a single subfamily compared to those across different subfamilies. Variations in exon counts and intron distribution were observed among members, while gene structures within each subfamily exhibited high homology. Computational analysis of conserved motifs revealed that all members shared identical motifs (motif 1, 2, and 3), suggesting similar functions. The synergistic differentiation between conserved motifs and gene structure reflects the balance between functional conservation and diversity during the evolution of the TaNF-YB family.

Chromosomal mapping analysis (Figure 2C) shows that the 34 *TaNF-YBs* are unevenly distributed across 15 chromosomes: Chr1A/B/D, Chr2A/B/D, Chr3D, Chr6A/B/D, and Chr7B/D each contain two *TaNF-YBs*; Chr3A/B each contain three; and Chr7A contains four *TaNF-YBs*.

### 2.3. Collinearity Analysis of the TaNF-YBs

To investigate the collinearity relationships among *TaNF-YBs*, we employed the Advanced Circos plugin in TBtools-II v2.322 software for analysis. The computationally estimated results revealed an uneven distribution of *TaNF-YBs* across the A/B/D subgenomes of seven homologous gene groups in wheat; fragment-replication genes were predominant, while tandem-replication genes were minority (Figure S2). These findings indicate that fragment replication serves as the primary driver of TaNF-YB family expansion. To assess the evolutionary constraints on homologous gene pairs, researchers calculated Ka and Ks values; all *TaNF-YB* homologous gene pairs exhibited Ka/Ks values less than 1, suggesting strong purifying selection during evolution (Table 2).

**Figure 3:**
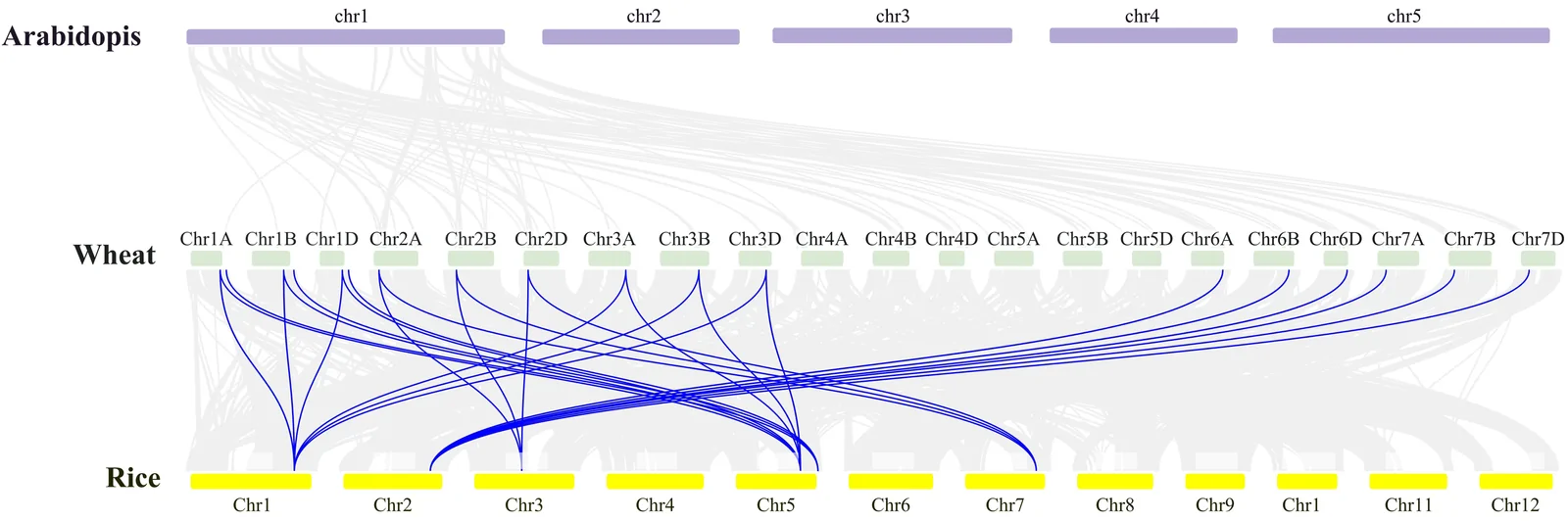
Predicted synteny analysis of NF-YBs across wheat, Arabidopsis, and rice genomes. The light gray lines represent the collinear blocks in wheat vs. Arabidopsis and wheat vs. rice. The syntenic *TaNF-YBs* pairs are shown connected by the blue lines.

To elucidate the evolutionary mechanisms of the wheat NF-YB gene family, this study constructed cross-species homology alignment maps comparing *Arabidopsis thaliana*, rice, and wheat. Predicted results (Figure 3) revealed 27 identified NF-YB homologous gene pairs between wheat and rice genomes, including multiple instances where wheat NF-YBs correspond to identical rice homologs, reflecting species-specific expansion during the wheat heteroploidization process. In contrast, no NF-YB homologous gene pairs were detected between wheat and Arabidopsis—this is likely attributable to early evolutionary divergence between monocotyledonous and dicotyledonous plants, which led to significant rearrangement of genomic homology regions and hindered the effective identification of homologous sequences.

### 2.4. Analysis of Cis-Acting Elements in the Promoter of TaNF-YBs in Wheat

**Figure 4:**
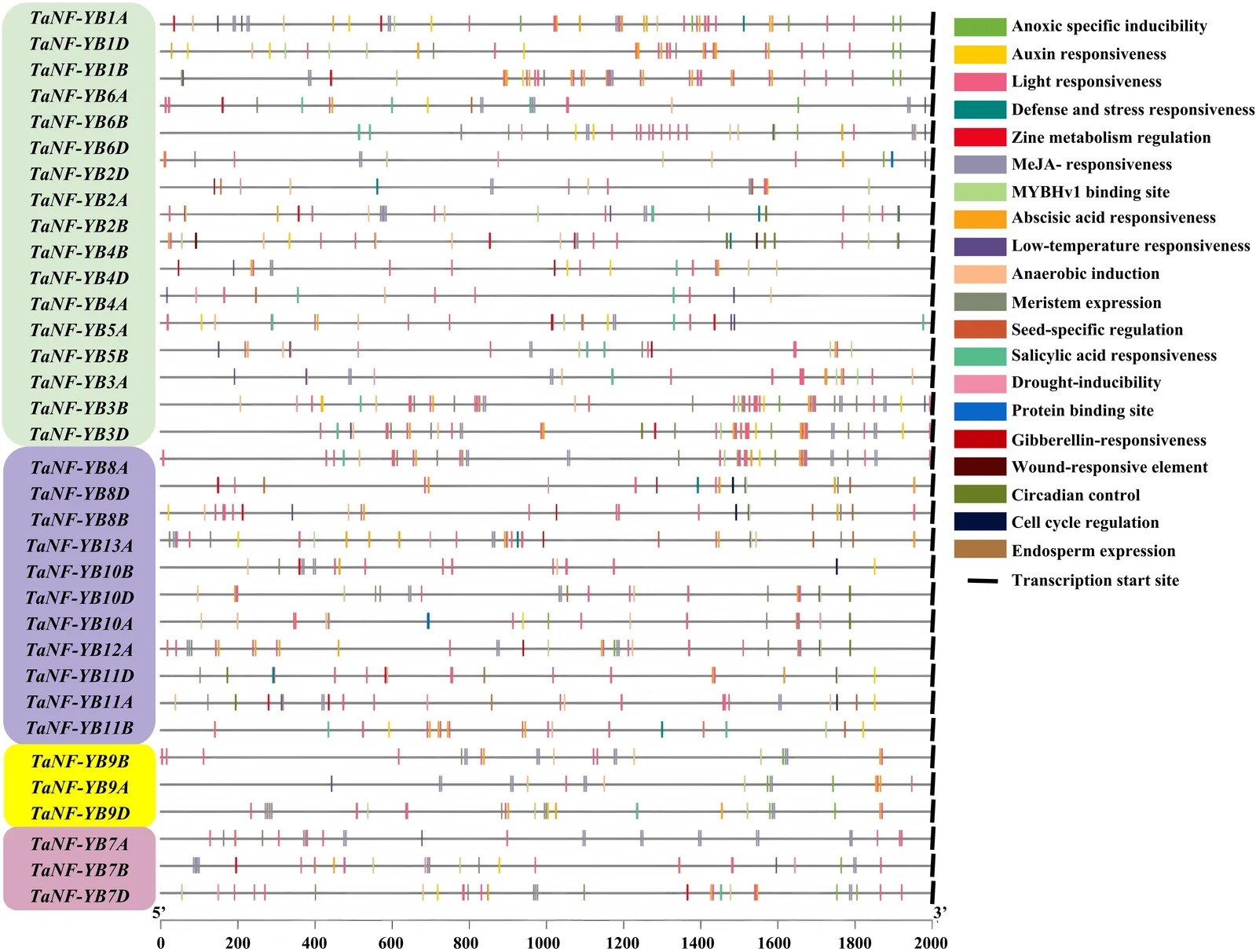
Predicted Cis-acting regulatory elements in the promoter regions of *TaNF-YBs*. Different colors indicate different cis-acting regulatory elements.

To investigate the potential functions of *TaNF-YB* elements, the research team utilized the TBtools tool to extract 2000 bp upstream sequences from 34 *TaNF-YBs* transcription initiation sites in the wheat genome and analyzed cis-regulatory elements using the online PlantCARE database. A number of hormone-responsive elements—including methyl jasmonate, abscisic acid, salicylic acid, auxins, and gibberellins—as well as stress-related elements associated with low temperature, drought, anaerobiosis, disease resistance, and injury, were predicted. Additionally, numerous developmental regulatory elements were detected, covering light-responsive, meristem development, endosperm function, seed development, cell cycle, and circadian rhythms (Figure 4 and Table S2). Light-responsive elements (295) constituted the largest categories, followed by MeJA-related elements (168), ABA-related elements (139), and anaerobic induction elements (61). Elements related to drought, biological stress, and cell cycle accounted for a smaller proportion. Significant variations were observed in both the diversity and quantity of elements among different *TaNF-YB* members: *TaNF-YB1B*, *TaNF-YB3A*, and *TaNF-YB3B* exhibited enrichment across multiple hormone- and light-responsive cis-elements, whereas certain genes contained fewer elements across all categories. These predictions suggest that distinct copies of the wheat TaNF-YB family may exhibit functional specialization in hormone signaling, abiotic stress responses, and plant growth and development processes, enabling comprehensive regulation through integrated internal and environmental signals.

### 2.5. Analysis of TaNF-YBs Expression Pattern Based on RNA-Seq

Following sequence quality control and filtering, the resulting valid reads presented generally high sequencing quality. Across all experimental samples, the percentage of bases with a Phred quality score greater than 30 (Q30) exceeded 95%, and the GC content remained stably maintained at approximately 54%, which verified that the obtained transcriptome sequencing data had high reliability (Table S3).

**Figure 5:**
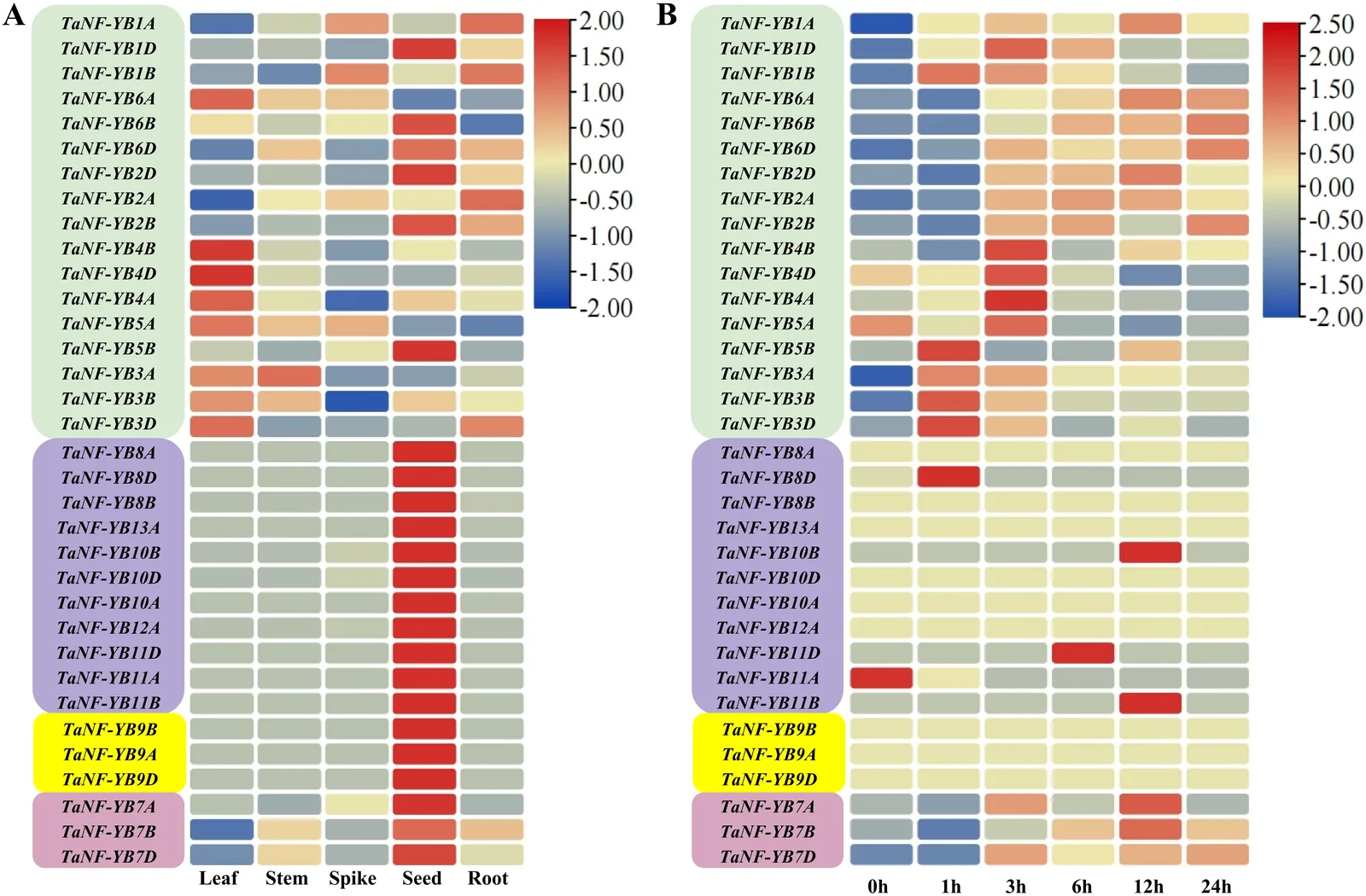
Expression patterns of *TaNF-YBs*. (**A**) A heatmap of *TaNF-YBs* in leaf, stem, spike, seed, and root. (**B**) Analysis of expression patterns of *TaNF-YBs* under salt stress at different time points.

To investigate the function of *TaNF-YBs*, we analyzed the expression profiles of 34 *TaNF-YBs* across different tissues. The results (Figure 5A, Table S4) showed that TaNF-YB family genes are predominantly highly expressed in seeds, indicating that this family may participate in the processes of wheat seed development, grain filling, and storage substance synthesis. Specific genes exhibited low expression levels in leaves and spikes, such as *TaNF-YB1A*, *TaNF-YB2A*, and *TaNF-YB7B* in leaves and *TaNF-YB4A* and *TaNF-YB3B* in spikes. Expression of the TaNF-YB family remained relatively stable in stems.

To elucidate the expression response patterns of *TaNF-YBs* under salt stress, an expression heatmap was constructed based on short-term time-series transcriptomic data. The results (Figure 5B, Table S5) revealed significant spatiotemporal differences in expression among subfamilies under salt stress, exhibiting diverse response profiles: *TaNF-YB1D*, *TaNF-YB4B*, *TaNF-YB4D*, *TaNF-YB4A*, and *TaNF-YB5A* showed rapid upregulation during early salt stress (1–3 h); *TaNF-YB11D* reached peak expression at 6 h, while *TaNF-YB10B* and *TaNF-YB11B* peaked at 12 h; all *TaNF-YBs* exhibited a gradual decline in expression by 24 h post-salt stress. The expression profile of the *TaNF-YBs* exhibits distinct differentiation under salt stress, indicating that different members of this family may perform distinct regulatory functions in wheat response to salt stress and providing a reference for further investigation of NF-YB salt-tolerant genes.

### 2.6. RNA-Seq and qRT-PCR Validation

**Figure 6:**
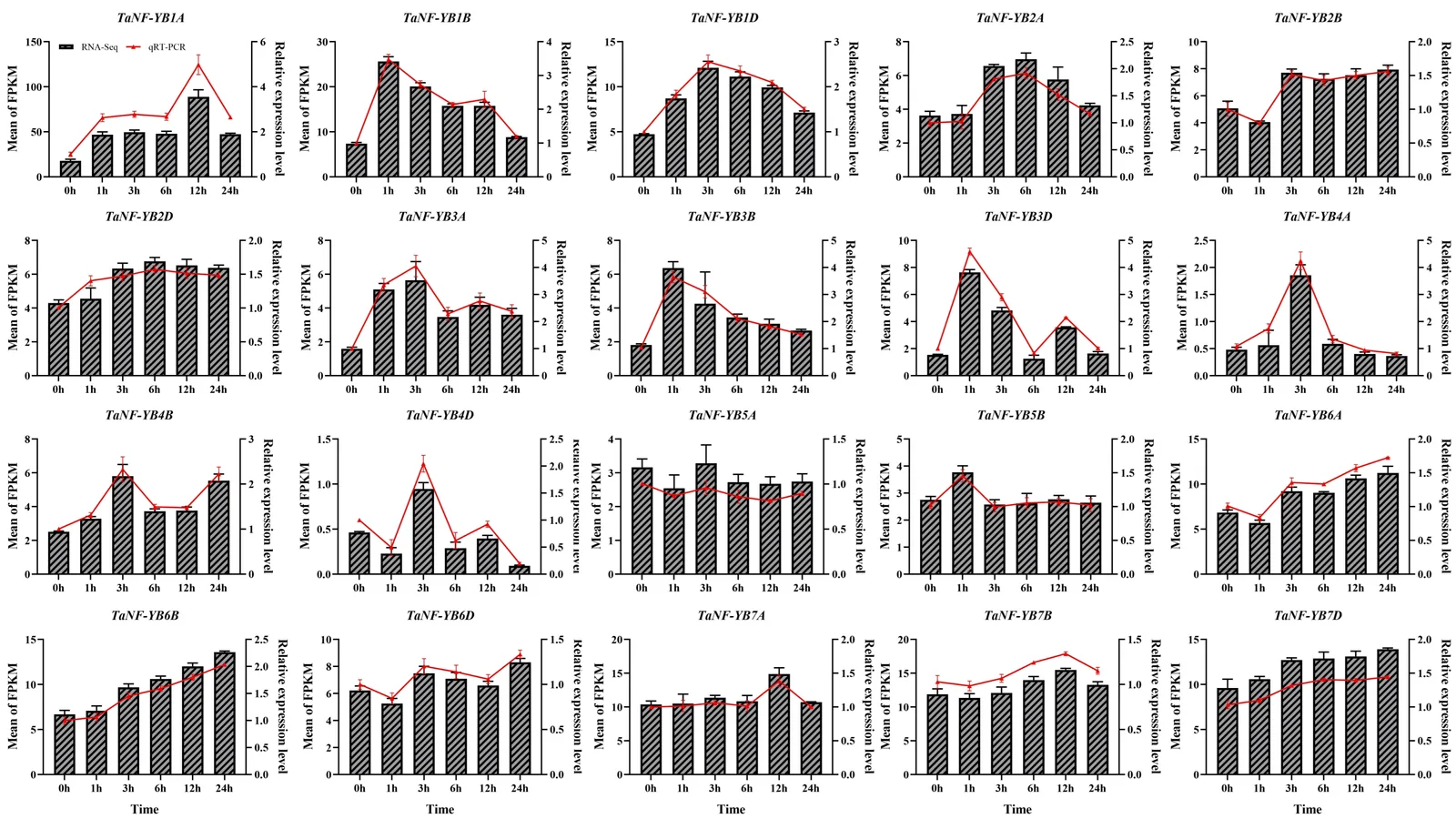
RNA-Seq was validated by qRT-PCR. Black bars represent the mean FPKM values from RNA-Seq data; red lines indicate relative expression levels obtained by qRT-PCR. Relative expression levels were calculated using the 2^−ΔΔCt^ method with *TaActin* as the internal reference. The X-axis represents salt-stress treatment time points (0 h, 1 h, 3 h, 6 h, 12 h, and 24 h). Each error bar indicates the standard deviation from three biological replicates. Plant material: Wheat cv. Pinyu 8155.

Through comprehensive analysis of gene expression profiles under salt stress in wheat, we identified 20 genes with statistically significant differential expression as key genes. To validate these findings, we quantified the expression levels of these 20 genes using qRT-PCR. The results demonstrated that their expression patterns in strain Pinyu 8155 were consistent with those obtained from RNA-seq data (Figure 6, Tables S4 and S6), thereby confirming the reliability of the transcriptomic analysis methodology.

### 2.7. GO Enrichment Analysis of TaNF-YBs

**Figure 7:**
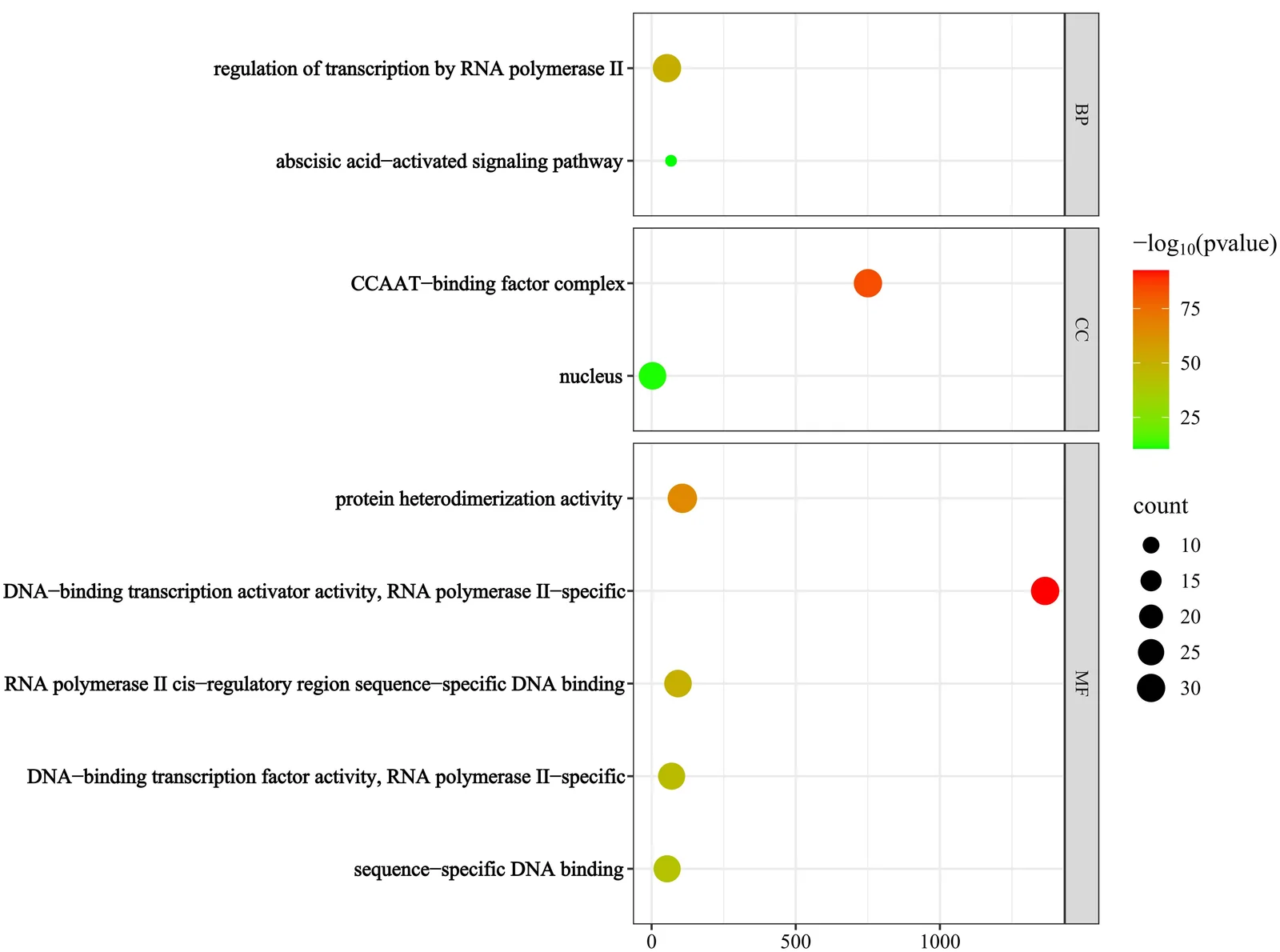
GO enrichment analysis of *TaNF-YBs*.

To elucidate the molecular mechanisms by which the TaNF-YB gene participates in plant responses to salt stress, a GO functional enrichment analysis was conducted (Figure 7 and Table S7). The results demonstrated that the functional enrichment of this gene family primarily focused on DNA-binding transcription activator activity, RNA polymerase II-specificity, CCAAT-binding factor complexes, and protein heterodimerization activity, all of which exhibited highly significant enrichment levels (*p*-value <0.01). In biological processes, *TaNF-YBs* were markedly enriched in RNA polymerase II-mediated transcriptional regulation and abscisic acid-activated signaling pathways. Cellular component enrichment analysis suggests that the TaNF-YB protein is primarily localized in the nucleus and exhibits significant enrichment in the CCAAT-binding factor complex. These findings confirm that TaNF-YB, as a nuclear transcription factor, can participate in the transcriptional regulation of target genes through the assembly of functional complexes. At the molecular function level, these genes were significantly enriched in core processes including protein heterodimerization, sequence-specific DNA binding, RNA polymerase II-specific transcriptional activation, and cis-regulatory region binding. This indicated that TaNF-YB proteins may assemble functional complexes via protein–protein interactions to bind downstream salt-tolerance target gene promoters and activate their transcriptional expression. In conclusion, *TaNF-YBs* family members may positively regulate salt-tolerance-related gene expression via nuclear transcriptional regulation, contributing to wheat response to salt stress.

### 2.8. Analysis of Protein–Protein Interactions of TaNF-YBs

**Figure 8:**
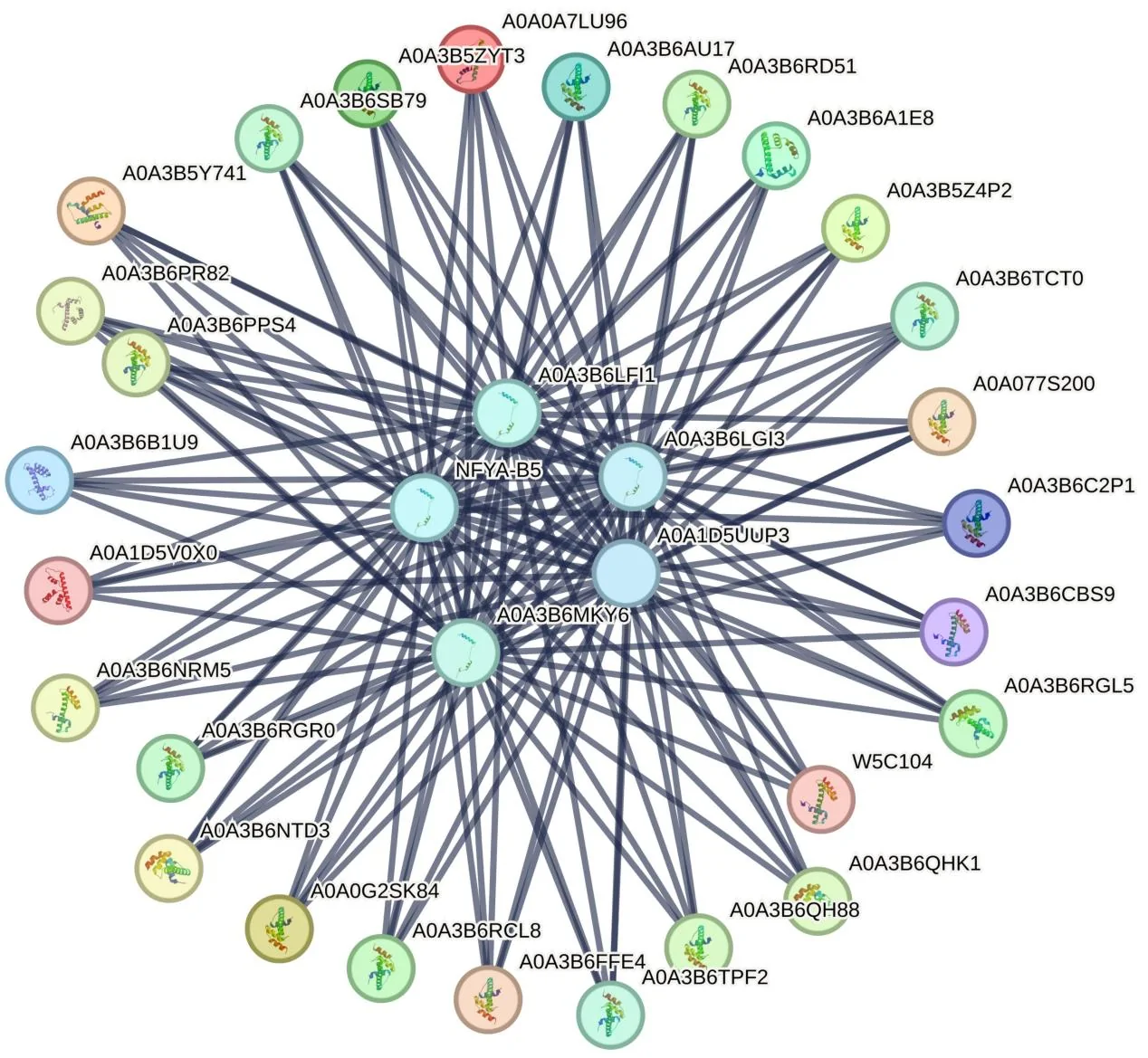
Predicted protein–protein interactions network of TaNF-YB proteins.

A predicted protein–protein interaction network of TaNF-YB proteins was constructed using STRING (Figure 8 and Table S8). Of the 34 TaNF-YB proteins, 27 met the 0.9 confidence threshold and were retained in the visualization, while the remaining 7 were excluded due to a lack of eligible interaction pairs. All 27 interacting NF-YB proteins demonstrated broad-binding capacity to both the network center NF-YA (NFYA-B5) and multiple NF-YC subtypes, consistent with the heterotrimer assembly pattern of NF-Y proteins. The absence of interactions among the seven non-interacting NF-YB subtypes in predicted trimer complexes suggests functional specialization: these subtypes may independently perform transcriptional regulation or specifically interact with other partner proteins under specific stress conditions or developmental stages.

## 3. Discussion

### 3.1. Genome-Wide Identification of NF-YB Gene Family in Wheat

The NF-YB gene family comprises transcription factors that regulate plant responses to stress conditions. Through bioinformatics identification of the NF-YB genes and analysis of their physicochemical properties and structural characteristics, this study facilitates a deeper understanding of NF-YB functions in plants and lays the foundation for further investigations into the family stress resistance mechanisms.

This study identified a total of 34 *TaNF-YBs*. The number of NF-YB members in wheat exceeds those found in rice [39], maize [42], and Arabidopsis [13], this phenomenon can be largely attributed to the polyploid characteristics of the wheat genome and its segmental duplication events. The strong purifying selection on the NF-YB domain reflects stringent functional constraints on TaNF-YB proteins as core subunits of the NF-Y transcription complex. Two sets of universally conserved residues underpin key properties of TaNF-YBs. Conserved interface cysteines act as redox-sensitive switches regulating NF-YB/NF-YC heterodimerization via reversible disulfide bonds, a mechanism conserved across fungi and plants [43,44], implying wheat NF-Y complexes may dynamically tune transcriptional activity in response to cellular redox status under stress. The conserved Arg-Asp salt bridge, a canonical histone fold motif stabilizer, anchors the L2 loop to the α3 helix to maintain proper HFM folding and stable subunit interaction [44]. The high evolutionary conservation of these functional residues indicates that TaNF-YBs retain deeply conserved roles in wheat growth, development, and stress adaptation. The average amino acid length of TaNF-YBs was approximately 226 residues, with an average molecular weight of 24 kDa (Table 1), consistent with previous findings [45]. Chromosomal mapping analysis revealed an uneven distribution of TaNF-YB family genes across chromosomes, with members of the same subfamily dispersed on different chromosomes, a pattern also observed in other crops [46,47]. Furthermore, in silico prediction indicated that all proteins of this family are likely localized in the nucleus, which is in agreement with related reports in rice [48] and maize [16].

The subfamily classification of TaNF-YBs was based on the NJ phylogenetic tree with 1000 bootstrap replicates. The results showed that all NF-YBs could be grouped into five evolutionary clades. Each clade contained NF-YB members from Arabidopsis and rice, whereas clades V lacks TaNF-YB members, indicating differences in the species-divergence time of these genes. Gene structure analysis shows that *TaNF-YBs* contain fewer introns: 15 genes including *TaNF-YB4B* lack introns entirely, while five genes such as *TaNF-YB5A* possess only one intron. This suggests that *TaNF-YBs* have undergone intense evolutionary selection pressure leading to substantial intron loss, a mechanism that stabilizes gene structure, reduces alternative splicing during transcription, enhances CCAAT-box-mediated transcription initiation, and accelerates transcriptional activation/inhibition responses, thereby improving environmental stress adaptation [49]. Conservation motif predicted analysis demonstrates that all 34 TaNF-YBs share Motif1, Motif2, and Motif3 motifs, indicating functional similarity among these genes. The central regions of certain NF-YB genes house their primary conserved domains, whose positional conservation enables NF-YB proteins to form dimers via binding with NF-YC proteins. These dimers subsequently interact with NF-YA proteins to constitute functional heterotrimeric nuclear transcription factors [50].

Predicted collinear gene pairs within the TaNF-YB gene family were predominately derived from fragment duplication events, suggesting that this process serves as the primary mechanism driving the expansion of the TaNF-YB family (Figure S2). Compared with analyses between Arabidopsis and wheat, the comparison between wheat and rice reveals significantly more collinear gene pairs (Figure 3), likely reflecting their closer phylogenetic relationship as members of the Poaceae monocotyledonous plants. In contrast, no NF-YB homologous gene pairs were detected between wheat and Arabidopsis, probably due to significant rearrangements in genomic homologous regions during early evolutionary divergence between monocots and dicots, which hindered effective identification of homologous sequences, a finding also observed in the YABBY gene family [51].

Cis-acting elements are the core components regulating the spatiotemporal specificity of gene expression. In TaNF-YB promoter regions, computationally predicted elements include a large set of light-responsive elements (295); hormone-responsive elements including MeJA (168) and ABA (139); and elements responsive to abiotic stresses such as low temperature, drought, hypoxia, defense response, and wounding. The distribution patterns of these predicted cis-regulatory elements are consistent with the established roles of the NF-Y transcription factor family in mediating abiotic stress responses through the ABA/MeJA signaling pathway and further provide preliminary computational evidence supporting the putative involvement of *TaNF-YBs* in plant salt-stress response [31,52,53,54,55]. Light-responsive elements represented the most abundant category. In addition, developmental regulatory elements were also predicted, including those involved in meristem development, endosperm function, seed development, cell cycle, and circadian rhythm. These findings align with the reported functions of *LEC1* (*NF-YB9*) in regulating embryonic and seed development, and of *TaNF-YB3* in modulating photosynthetic genes [2,56,57,58]. The diversity and copy numbers of predicted cis-elements varied markedly among different *TaNF-YBs*. *TaNF-YB1B*, *TaNF-YB3A*, and *TaNF-YB3B* displayed enriched element profiles, whereas some genes showed relatively low abundance across all elements categories. This suggests that individual gene copies may have undergo subfunctionalization or neofunctionalization following polyploidization, giving rise to specialized regulatory modules [5,39]. In summary, distinct copies of the TaNF-YB gene family may achieve functional specialization by integrating hormonal, stress, and developmental signals, with several members potentially participating in salt stress responses. Notably, the PlantCARE-based analysis represents sequence-level computational prediction, the presence of a candidate element does not guarantee its functional activity, and thus follow-up in vivo experiments are required to validate the regulatory roles of key element.

### 3.2. Expression Profiles and Predicted Protein–Protein Interaction Analysis of TaNF-YBs

In this study, the expression profiles of *TaNF-YBs* across different tissues and under varying temporal salt stress were comprehensively analyzed, using RNA-seq and qRT-PCR datas. qRT-PCR validation exhibited overall correlation with RNA-seq derived expression trends, supporting the reliability of transcriptome-based expression patterns for TaNF-YB family members. The consistency between the two datasets reinforced the tissue-specific and time-dependent transcriptional reprogramming of *TaNF-YBs* upon developmental and salinity stimuli, which provides a solid basis for inferring their potential biological functions from expression characteristics.

Tissue transcriptomic data retrieved from public databases revealed that *TaNF-YBs* were predominantly expressed in seeds, exhibit stable expression in stems, and show low abundance expression in leaves and spikes; this tissue-specific expression pattern aligns closely with findings from functional studies on NF-YBs in Poaceae [59]. This divergent tissue-preferential expression indicates functional partitioning within this gene family: seed-high-expressed members are mainly responsible for reproductive development and grain nutrient accumulation, while stably stem-expressed copies may participate in the vegetative growth and stress adaptation of above-ground tissues. A recent review shows that plant NF-Y transcription factors integrate endogenous signals like ABA and gibberellin to regulate embryogenesis, grain development and phase transition, which supports the biological significance of the seed-enriched expression pattern detected in this study [60]. LEC1-type homologous genes such as *OsNF-YB1* and *OsNF-YB9* are specifically highly expressed in the endosperm–pericarp layer, directly regulating grain filling and starch synthesis; their knockout leads to phenotypic defects including seed shriveling and impaired starch accumulation, further confirming NF-YBs role may as a core regulatory factor in cereal seed development [61,62]. The high-expression profile of wheat homologous *TaNF-YBs* in seeds may also underscore their conserved functions in grain formation and nutrient accumulation. However, functional studies are needed to confirm this possibility.

The temporal expression heatmap of *TaNF-YBs*, constructed using short-term time-series salt-stress transcriptomic data from wheat cultivar Pinyu 8155, showed that different members of this gene family exhibited markedly differentiated temporal response patterns under salt stress. Specifically, some members were rapidly upregulated in the early stage of salt stress (1–3 h), while the expression of other members peaked at 6 h or 12 h, and the overall expression level decreased after 24 h of stress treatment. This distinct temporal response pattern is a typical regulatory strategy adopted by plant transcription factor families to adapt to salt stress, which enables the precise regulation of downstream stress response cascades at different stress stages [63]. In finger millet, the qRT-PCR-based expression profiling of NF-Y genes under NaCl stress also demonstrated that *EcNF-YB6* and *EcNF-YB10* were significantly induced at the early stress stage (6 h). Phylogenetic analysis further confirmed that finger millet NF-YB genes have close evolutionary and functional associations with NF-YB members that regulate abiotic stress responses in wheat, rice, sorghum and maize [63]. Previous studies on the single-gene functions of wheat *TaNF-YB3* and *TaNF-YB2* similarly revealed that salt stress induces transient gene activation followed by sustained expression decline, with different copy numbers working synergistically to facilitate osmotic stress adaptation [64,65].

In conclusion, these tissue-specific expression patterns and dynamic temporal profiles of stress response indicate that members of the TaNF-YB gene family may participate in two core biological processes, namely seed developmental programming and dynamic salt stress signal transduction.

GO enrichment analysis provided functional evidence for the molecular mechanisms by which the TaNF-YB family regulates the salt stress response in wheat, with significant enrichment observed across the molecular function, cellular component, and biological process categories (*p* < 0.01). They were enriched in protein heterodimerization, sequence-specific DNA binding, RNA polymerase II-dependent transcriptional activation, and cis-regulatory region binding, consistent with NF-Y transcription factors assembling heterotrimers with NF-YA and NF-YC subunits to recognize CCAAT boxes [3,5], as *TaNF-YB2* does with *TaNF-YA7* and *TaNF-YC7* to activate stress defense genes [65]. They were predominantly nuclear and enriched in the CCAAT-binding factor complex [39] and in transcriptional regulation and abscisic acid signaling, indicating a transcriptional regulatory role in salt stress. These findings agree with salt-stress upregulation of *TaNF-YB* members in wheat [66], early induction of *CaNF-YB* members in pepper [67], and the strong NF-YB response in larch [68]. However, NF-YB functions vary across stresses, as *ZmNF-YB10* is upregulated under drought and salt stress but downregulated under alkaline and low-temperature stress [69]. Thus, individual *TaNF-YB* members may play distinct roles, requiring validation via transgenic analyses.

The predicted protein–protein interaction network analysis visually illustrates the canonical assembly pattern of TaNF-YB complexes: the vast majority of TaNF-YB proteins were predicted to interact extensively with diverse TaNF-YA and TaNF-YC subunits to form an intact NF-Y heterotrimer complex, representing a conserved functional mechanism to NF-Y transcription factors in eukaryotes. Trimer assembly follows a strict sequential process: NF-YB and NF-YC first form a cytoplasmic heterodimer through their histone fold motifs, translocate to the nucleus, and then recruit NF-YA to form a functional trimer. Only the complete trimer possesses high-affinity DNA binding capacity, where NF-YA recognizes the CCAAT box and the NF-YB/NF-YC heterodimer stabilizes this interaction [8,59,60]. Conserved motifs within the NF-YB domain directly mediate interactions between the NF-YB and NF-YA/NF-YC subunits as well as DNA binding, and these motifs are broadly conserved across Poaceae species including *Eleusine coracana*, *Sorghum bicolor* and *Setaria italica* [70]. These extensive interaction capabilities are largely consistent with the dual biological roles of TaNF-YBs indicated by expression profiling. Experimental evidence from *Phalaenopsis* has confirmed that NF-YB/NF-YC interactions directly modulate downstream gene expression: yeast two-hybrid and bimolecular fluorescence complementation assays verified the physical interaction between *PhNF-YB6* and *PhNF-YC7*, and transient overexpression of this pair promoted *PhFT3* expression and repressed *PhSVP* expression [71]. Diverse subunit combinations expand the target gene repertoire and enable integration of developmental and environmental signals.

In this study, seven TaNF-YB subtypes were identified in the predicted interaction network with no significant predicted interaction events. This phenomenon has been previously reported in NF-Y interaction studies of Arabidopsis [72] and maize [42]. It is hypothesized that these members with no detectable interaction partners may exert independent transcriptional regulatory functions, either autonomously or synergistically with other non-NF-Y proteins, thereby expanding the functional diversity of the NF-YB family [48,73,74].

Through integrated analysis of gene expression profile, functional enrichment and predicted protein–protein interaction networks, this study suggests that TaNF-YBs in wheat may play a dual role in regulating seed development and response to salt stress. These genes may enable the integration of multiple pathways through subfunctional differentiation and interactions with diverse proteins, which provides theoretical support for the screening and identification of candidate genes for breeding salt-alkali-tolerant, high-yield and high-quality grain.

### 3.3. Limitations and Future Prospects of This Study

Despite the systematic genome-wide characterization of the TaNF-YB family in this study, several limitations remain. First, all functional inferences were predominantly derived from bioinformatic analyses, including promoter cis-acting element prediction, GO enrichment, transcriptomic profiling, and in silico protein–protein interaction networks, which only provide preliminary predictive evidence. Notably, the chromatin-level regulatory mechanisms of TaNF-YB complexes (e.g., chromatin conformational remodeling, responses to intranuclear ionic shifts, and chromatin folding dynamics) remain unelucidated, and the putative salt-tolerance roles of *TaNF-YBs* require experimental validation. Second, expression profiling across limited tissues and sampling time points fails to fully capture the spatiotemporal dynamics of *TaNF-YBs* in response to salt stress. Furthermore, this study does not distinguish whether expression alterations reflect stable epigenetic remodeling or transient stress-triggered signaling events, leaving their long-term biological significance unaddressed. Third, functional redundancy among *TaNF-YB* members and within the entire NF-Y family hinders the dissection of isoform-specific functions.

Future investigations will focus on the following directions: First, high-resolution short-term time-series transcriptomics combined with epigenomic assays will be performed to delineate the full temporal dynamics of stress responses and disentangle the regulatory nature of expression changes. Second, molecular and biochemical assays (including ChIP-seq, protein structural simulation, nucleosome binding assays, and EMSA) will be employed to decipher the chromatin-level regulatory mechanisms and transcriptional activity of TaNF-YB complexes, while yeast two-hybrid and BiFC assays will validate NF-Y trimers assembly. Third, *in planta* genetic manipulation (overexpression, gene editing, and knockdown) will establish the causal relationship between *TaNF-YB* genes and salt-tolerance phenotypes. Finally, haplotype and association analyses using diverse wheat germplasm will support the development of functional markers for marker-assisted breeding.

## 4. Materials and Methods

### 4.1. Plant Materials and Treatments

The Pinyu 8155 variety is provided by the Grain Quality Laboratory of the Wheat Research Institute at Shanxi Agricultural University. It was approved nationally in 2019 (National Approval No. Guo Shen Mai 20190044) and is currently one of the leading dryland wheat varieties promoted in Shanxi Province [75].

In this study, Pinyu 8155 plants were subjected to salt-stress treatment, with three biological replicates per treatment group; each replicate sample was a pooled sample collected from 5 to 8 independent plants. The salt-stress treatment was applied by completely replacing the original hydroponic nutrient solution with fresh nutrient solution containing 150 mmol·L^−1^ NaCl [76,77]. Time-gradient salt-stress treatments were set as 0, 1, 3, 6, 12, and 24 h (T0, T1, T3, T6, T12, and T24). The cultivation conditions were as follows: daytime temperature 22 °C, nighttime temperature 18 °C, relative humidity 60%, light cycle of 16 h illumination/8 h darkness. After salt-stress treatment, the plant roots were rinsed with sterile water, immediately frozen in liquid nitrogen, and stored at –80 °C. The samples were subsequently sent to Jiangsu Sanshu Biotechnology Co., Ltd (Nantong, Jiangsu, China). (https://sanshugroup.com/) for transcriptome sequencing.

Prior to cDNA library construction, RNA integrity and purity were assessed, and library construction started with ≥1 μg of total RNA. mRNA was enriched by Oligo(dT) magnetic beads with an NEB kit (Ipswich, MA, USA), followed by fragmentation, cDNA synthesis, end repair, A-tailing, adapter ligation and PCR amplification. After construction, library quality and concentration were detected by Agilent 2100 Bioanalyzer (Santa Clara, CA, USA), PicoGreen assay (Thermo Scientific, Waltham, MA, USA) and quantitative PCR(Thermo Scientific, Waltham, MA, USA); qualified libraries were pooled and sequenced on Illumina platform in PE150 mode. Raw reads were filtered by Trimmomatic v0.36 to remove reads with base quality <20. Clean reads were aligned to wheat reference genome IWGSC RefSeq v2.1 by STAR. Transcripts were assembled by StringTie v2.1.6, and novel transcripts were identified by Gffcompare via comparison with reference annotation. Novel genes were annotated by DIAMOND Blastp alignment to Nr, Swiss-Prot and KOG databases. Differentially expressed genes were screened by DESeq2 with default normalization, thresholds were FDR < 0.05 and |log2(fold change)| ≥ 1.

### 4.2. Genome-Wide Identification of TaNF-YBs

The reference genome assembly of wheat (*Triticum aestivum* L.) cv. Chinese Spring (IWGSC v2.1) was downloaded from Phytozome13 (https://phytozome-next.jgi.doe.gov/, accessed 10 April 2026). The conserved NF-YB domain (PF00808) was validated using canonical *Arabidopsis thaliana* NF-YB sequences from the TAIR10 database (https://www.arabidopsis.org/, accessed 10 April 2026) using NCBI BLASTp (Bethesda, MD, USA) (https://blast.ncbi.nlm.nih.gov/Blast.cgi, accessed 11 April 2026).

The PF00808 hidden Markov model (HMM) profile was retrieved from Pfam (https://www.ebi.ac.uk/interpro/entry/pfam/#table, accessed 12 April 2026), and genome-wide screening for *TaNF-YB* candidates was performed using HMMER 3.0 with an E-value cutoff of 0.01. Redundant transcript isoforms were filtered through the Phytozome portal (https://data.jgi.doe.gov/refine-download/phytozome, accessed 13 April 2026), retaining only the primary transcript per gene.

Full-length TaNF-YB protein sequences were extracted with TBtools and curated for integrity. Physicochemical parameters (amino acid length, molecular weight, theoretical isoelectric point) were calculated via ExPASy ProtParam (https://web.expasy.org/protparam/, accessed 15 April 2026), and subcellular localization was predicted using the Plant-mPLoc server (http://www.csbio.sjtu.edu.cn/bioinf/plant-multi/#, accessed 16 April 2026).

### 4.3. Conserved Domains, Phylogenetic Tree Construction and Chromosome Localization

To investigate the evolutionary characteristics and phylogenetic relationships of TaNF-YB proteins in Arabidopsis, rice, and wheat, we conducted a series of bioinformatics analyses. First, we aligned TaNF-YB protein sequences using Clustal W in MEGA 7.0 software and visualized the results with Jalview to identify conserved regions. Second, we obtained NF-YB protein sequences from rice and Arabidopsis in the Plant TFDB database (https://plantrnadb.com/wheatrna/, accessed on 20 April 2026); performed multisequence alignment of NF-YB full-length sequences from wheat, Arabidopsis, and rice using Clustal W in MEGA11(v11.0.13); constructed a phylogenetic tree via the neighborjoining (NJ) method (with bootstrap value set to 1000); and visualized and classified the tree using iTOL v5 (https://itol.embl.de, accessed on 21 April 2026). Finally, we performed chromosomal localization analysis of the *NF-YB* gene family with TBtools-II v2.322.

### 4.4. Gene Structure and Conserved Motif Prediction

The gene structure of TaNF-YBs were analyzed using the GFF annotation file of the wheat genome. Conserved-motif analysis was performed on the TaNF-YB protein sequences via the MEME online platform (https://meme-suite.org/meme/tools/meme, accessed on 25 April 2026), with the maximum number of motifs set to 10. TBtools software was used for the visualization of gene structures and conserved motifs.

### 4.5. Collinearity Analysis

A collinearity analysis was performed on *TaNF-YBs* using the MCScanX plugin in TBtools for both collinearity assessment and Circos plot generation. The CDS of the NF-YB gene family were pairwise aligned using NCBI Blast online platform (https://blast.ncbi.nlm.nih.gov/Blast.cgi, accessed on 28 April 2026), with the Simple Ka/Ks Calculator plugin calculating Ka/Ks ratios. Subsequently, the Multiple Synteny Plot tool in TBtools was employed to analyze and visualize collinearity relationships among wheat, rice, and Arabidopsis thaliana.

### 4.6. Analysis of Cis-Acting Elements in Promoter Regions

Using the TBtools software, we obtained the 2000 bp upstream sequences from the transcription initiation sites of wheat NF-YB gene. Promoter cis-regulatory elements were predicted via the PlantCARE website (https://bio.tools/plantcare, accessed on 7 May 2026). TBtools was further used to visualize the prediction results.

### 4.7. Analysis of TaNF-YBs Expression Patterns

To elucidate the expression patterns of the TaNF-YB gene family in wheat, this study downloaded publicly available transcriptomic data from the wheat RNA-seq database (https://ipf.sustech.edu.cn/pub/wheatrna/, accessed on 8 May 2026), extracted FPKM expression values for each gene, and investigated their expression profiles across different wheat tissues. To further investigate the expression profile of the *TaNF-YBs* under salt stress, we conducted an analysis of short-term time-series salt-stress transcriptome data derived from Pinyu 8155, with FPKM values adopted as the metric for gene expression quantification. Differential expression analysis was performed at both the transcript level and the gene level using DESeq2 (v1.40.2), and a heatmap was constructed via TBtools to visually present the expression pattern of the *TaNF-YBs*.

### 4.8. Reverse Transcription Quantitative Polymerase Chain Reaction (RT-qPCR) Validation

To validate the reliability of RNA-seq results, this study screened 20 genes exhibiting significant expression differences under salt stress for further verification using qRT-PCR (Thermo Scientific, Waltham, MA, USA). cDNA was synthesized using the TaKaRa reverse transcription kit (Kusatsu, Shiga, Japan). With *TaActin* as the internal reference gene and SYBR Green I as the fluorescent dye, quantitative detection was performed on genes exhibiting significant expression differences under salt-stress conditions using the Applied Biosystems 7500 real-time PCR system (Thermo Scientific, Waltham, MA, USA). All reactions were conducted with three technical replicates; quantitative PCR primers were designed with Primer 5 software, with specific sequences detailed in Table S8; relative gene expression levels were calculated using the 2^−ΔΔCT^ method; and results are expressed as mean ± standard deviation (SD) [78].

### 4.9. GO Enrichment Analysis of the TaNF-YBs

To systematically elucidate the biological functions and mechanisms of the TaNF-YB gene family in wheat, this study employed the DAVID 6.8 online functional annotation database (https://davidbioinformatics.nih.gov/, accessed on 9 May 2026) for GO functional enrichment analysis. The enrichment analysis utilized the modified Fisher’s exact test to assess statistical significance, with a *p*-value < 0.01 serving as the threshold for highly significant enrichment. Ultimately, we identified significantly enriched GO functional entries for the TaNF-YBs.

### 4.10. Protein–Protein Interaction (PPI) Analysis of the TaNF-YBs

The interaction proteins of TaNF-YBs were predicted using the UniProt Protein Database 2026_01 (https://www.uniprot.org/, accessed on 9 May 2026) and STRING v11.5 (https://cn.string-db.org/, accessed on 9 May 2026). We set a high confidence threshold (combined score ≥ 0.9) to filter valid interaction relationships. To ensure network simplicity and clarity, isolated protein nodes without any interactions are automatically hidden by default.

## 5. Conclusions

The NF-YB transcription factor subfamily serves as a core regulator of plant growth, development, and abiotic stress responses. To date, systematic genome-wide characterization of this gene family in wheat remains limited. In this study, 34 *TaNF-YBs* were computationally predicted and systematically characterized at the genome-wide level using the latest wheat reference genome assembly and phylogenetically classified into four subfamilies. Evolutionary analysis inferred that fragment duplication was the primary mechanism driving the expansion of this family during wheat polyploidization, with pervasive purifying selection constraining all family members. Integrated analysis of expression profiles, predicted functional enrichment, and predicted protein–protein interaction networks suggests that the TaNF-YB family may integrate multiple signaling pathways through subfunctional differentiation and interactions with various proteins and exert putative dual regulatory functions in seed development and salt-stress responses. Collectively, these findings provide theoretical support for further dissection of the molecular mechanisms underlying wheat stress adaptation and lay a foundation for screening candidate genes for the molecular breeding of salt-alkali-tolerant, high-yield and high-quality wheat.

## Figures and Tables

**Table 1:** Basic information of predicted TaNF-YB proteins in wheat.

Gene Name	Gene ID	Genomic Location	CDS Length (bp)	Amino Acid Length (aa)	Molecular Weight (kDa)	Theoretical pI	Subcellular Localization
*TaNF-YB1A*	*TraesCS1A03G0725000.2*	Chr1A:486939949..486943754 reverse	549	182	19.01	5.26	Nucleus
*TaNF-YB2A*	*TraesCS1A03G1006100.1*	Chr1A:576104728..576106997 forward	411	136	14.93	5.65	Nucleus
*TaNF-YB1B*	*TraesCS1B03G0827200.1*	Chr1B:524193223..524197130 reverse	549	182	19.00	5.46	Nucleus
*TaNF-YB2B*	*TraesCS1B03G1187500.1*	Chr1B:671331249..671333579 forward	411	136	14.89	5.65	Nucleus
*TaNF-YB1D*	*TraesCS1D03G0689500.1*	Chr1D:387984268..387988290 reverse	549	182	19.00	5.45	Nucleus
*TaNF-YB2D*	*TraesCS1D03G0969000.1*	Chr1D:479185241..479187728 forward	411	136	14.97	5.37	Nucleus
*TaNF-YB3A*	*TraesCS2A03G0361500.1*	Chr2A:136252709..136253934 forward	681	226	23.40	5.74	Nucleus
*TaNF-YB4A*	*TraesCS2A03G0876600.1*	Chr2A:607222570..607223567 forward	750	249	26.20	6.25	Nucleus
*TaNF-YB3B*	*TraesCS2B03G0480700.1*	Chr2B:186419860..186421170 forward	678	225	23.30	5.74	Nucleus
*TaNF-YB4B*	*TraesCS2B03G0971400.1*	Chr2B:549624991..549626044 forward	750	249	27.18	6.41	Nucleus
*TaNF-YB3D*	*TraesCS2D03G0377200.1*	Chr2D:127655921..127657076 forward	678	225	23.37	5.74	Nucleus
*TaNF-YB4D*	*TraesCS2D03G0821400.1*	Chr2D:462947446..462948415 forward	750	249	28.18	6.49	Nucleus
*TaNF-YB5A*	*TraesCS3A03G0053500.1*	Chr3A:14344154..14345109 reverse	609	202	21.80	5.60	Nucleus
*TaNF-YB6A*	*TraesCS3A03G0840600.1*	Chr3A:689455389..689459896 forward	492	163	17.73	5.93	Nucleus
*TaNF-YB7A*	*TraesCS3A03G1044900.1*	Chr3A:601330110..601333216 reverse	876	291	31.48	4.92	Nucleus
*TaNF-YB5B*	*TraesCS3B03G0040400.1*	Chr3B:12369256..12370173 forward	639	212	22.66	5.79	Nucleus
*TaNF-YB6B*	*TraesCS3B03G0964500.1*	Chr3B:620690807..620693730 reverse	492	163	17.74	5.93	Nucleus
*TaNF-YB7B*	*TraesCS3B03G1206200.1*	Chr3B:748517538..748522333 forward	891	296	32.01	5.00	Nucleus
*TaNF-YB6D*	*TraesCS3D03G0771500.1*	Chr3D:459445001..459447892 reverse	492	163	17.74	5.93	Nucleus
*TaNF-YB7D*	*TraesCS3D03G0972900.1*	Chr3D:553696442..553700940 forward	897	298	32.16	5.07	Nucleus
*TaNF-YB8A*	*TraesCS6A03G0761500.1*	Chr6A:521570812..521571986 reverse	741	246	26.28	6.20	Nucleus
*TaNF-YB9A*	*TraesCS6A03G0761800.2*	Chr6A:521928124..521928902 reverse	516	171	18.43	6.16	Nucleus
*TaNF-YB8B*	*TraesCS6B03G0906300.1*	Chr6B:572435642..572436383 reverse	741	246	26.32	6.20	Nucleus
*TaNF-YB9B*	*TraesCS6B03G0906400.1*	Chr6B:572630147..572630937 reverse	492	163	17.74	6.17	Nucleus
*TaNF-YB8D*	*TraesCS6D03G0644500.1*	Chr6D:399275603..399276344 reverse	741	246	26.28	6.20	Nucleus
*TaNF-YB9D*	*TraesCS6D03G0644600.1*	Chr6D:399398757..399399615 reverse	492	163	17.67	6.17	Nucleus
*TaNF-YB10A*	*TraesCS7A03G0499700.1*	Chr7A:184640987..184642139 reverse	897	298	31.88	6.17	Nucleus
*TaNF-YB11A*	*TraesCS7A03G0500000.1*	Chr7A:185326414..185327308 reverse	801	266	28.69	5.02	Nucleus
*TaNF-YB12A*	*TraesCS7A03G0500200.1*	Chr7A:185645940..185647097 reverse	825	274	28.92	5.23	Nucleus
*TaNF-YB13A*	*TraesCS7A03G0500400.1*	Chr7A:186110979..186115472 reverse	801	266	27.58	4.88	Nucleus
*TaNF-YB10B*	*TraesCS7B03G0315100.1*	Chr7B:145173765..145174914 reverse	903	300	32.11	5.43	Nucleus
*TaNF-YB11B*	*TraesCS7B03G0316500.1*	Chr7B:146041504..146042664 reverse	843	280	29.70	5.21	Nucleus
*TaNF-YB10D*	*TraesCS7D03G0482700.1*	Chr7D:178390556..178391695 reverse	882	293	31.49	5.42	Nucleus
*TaNF-YB11D*	*TraesCS7D03G0483300.1*	Chr7D:178522880..178524035 reverse	894	297	31.54	5.19	Nucleus

**Table 2:** Computationally estimated Ka/Ks ratios and homologous gene pairs.

Homologous Genes		Ka	Ks	Ka/Ks	Homologous Fragment Length	Identities (%)
Gene1	Gene2					
*TaNF-YB1A*	*TaNF-YB1B*	0.00718	0.02413	0.29766	546	99.45
*TaNF-YB1A*	*TaNF-YB1D*	0.00719	0.04900	0.14666	546	99.45
*TaNF-YB1A*	*TaNF-YB6A*	0.13819	0.50618	0.27300	489	89.07
*TaNF-YB1A*	*TaNF-YB6B*	0.13510	0.48727	0.27726	489	89.07
*TaNF-YB1A*	*TaNF-YB6D*	0.13503	0.48832	0.27653	489	89.07
*TaNF-YB1B*	*TaNF-YB1D*	0.00478	0.05741	0.08335	546	99.45
*TaNF-YB1B*	*TaNF-YB6A*	0.13340	0.49828	0.26771	489	89.07
*TaNF-YB1B*	*TaNF-YB6B*	0.13033	0.47955	0.27177	489	89.07
*TaNF-YB1B*	*TaNF-YB6D*	0.13026	0.48058	0.27105	489	89.07
*TaNF-YB1D*	*TaNF-YB6D*	0.13819	0.54323	0.25438	489	89.07
*TaNF-YB2A*	*TaNF-YB2B*	0.00634	0.03359	0.18883	408	99.27
*TaNF-YB2A*	*TaNF-YB2D*	0.01273	0.10606	0.11999	408	99.27
*TaNF-YB2B*	*TaNF-YB2D*	0.00634	0.06875	0.09225	408	99.27
*TaNF-YB3A*	*TaNF-YB3B*	0.00784	0.06442	0.12168	675	99.12
*TaNF-YB3A*	*TaNF-YB3D*	0.01178	0.03158	0.37299	675	99.12
*TaNF-YB3B*	*TaNF-YB3D*	0.00390	0.03165	0.12334	675	99.56
*TaNF-YB4A*	*TaNF-YB4B*	0.01070	0.02796	0.38261	747	99.60
*TaNF-YB4A*	*TaNF-YB4D*	0.01430	0.02227	0.64240	747	99.60
*TaNF-YB4B*	*TaNF-YB4D*	0.01070	0.02796	0.38261	747	99.60
*TaNF-YB5A*	*TaNF-YB5B*	0.02023	0.12678	0.15959	588	96.55
*TaNF-YB6A*	*TaNF-YB6B*	0.00262	0.02867	0.09140	489	99.39
*TaNF-YB6A*	*TaNF-YB6D*	0.00262	0.00945	0.27717	489	99.39
*TaNF-YB6B*	*TaNF-YB6D*	−0.000	0.01899	0.00000	489	99.39
*TaNF-YB7A*	*TaNF-YB7B*	0.01228	0.05265	0.23328	873	99.66
*TaNF-YB7A*	*TaNF-YB7D*	0.00611	0.05772	0.10591	873	99.66
*TaNF-YB7B*	*TaNF-YB7D*	0.00602	0.06629	0.09079	888	99.66
*TaNF-YB8A*	*TaNF-YB8B*	0.00709	0.08660	0.08187	738	99.60
*TaNF-YB8A*	*TaNF-YB8D*	0.00354	0.06070	0.05830	738	99.60
*TaNF-YB8A*	*TaNF-YB10A*	0.41797	0.89177	0.46870	708	95.55
*TaNF-YB8B*	*TaNF-YB8D*	0.00710	0.04811	0.14750	738	99.60
*TaNF-YB8B*	*TaNF-YB10A*	0.37659	0.82779	0.45494	687	92.71
*TaNF-YB8D*	*TaNF-YB10A*	0.42012	0.85853	0.48934	708	95.55
*TaNF-YB9A*	*TaNF-YB9B*	0.01529	0.02838	0.53881	489	94.77
*TaNF-YB9A*	*TaNF-YB9D*	0.01672	0.03239	0.51620	489	94.77
*TaNF-YB9A*	*TaNF-YB9D*	0.00831	0.03248	0.25573	489	99.39
*TaNF-YB10A*	*TaNF-YB10B*	0.06912	0.16222	0.42608	894	99.67
*TaNF-YB10A*	*TaNF-YB10D*	0.06321	0.15114	0.41824	879	97.99
*TaNF-YB10B*	*TaNF-YB10D*	0.04367	0.09742	0.44822	879	97.34
*TaNF-YB11A*	*TaNF-YB11B*	0.04255	0.12211	0.34842	792	98.88
*TaNF-YB12A*	*TaNF-YB11D*	0.04273	0.08920	0.47904	822	99.64

## Data Availability

The original contributions presented in this study are included in the article/Supplementary Materials. Further inquiries can be directed to the corresponding authors.

## Supplementary Materials

The following supporting information can be downloaded at: https://www.mdpi.com/article/10.3390/ijms272110009/s1.
