# Supplementary material for: Genome-Wide Identification and Expression Analysis of the NF-YB Gene Family in Wheat (Triticum aestivum L.) Under Short-Term Salt Stress
Source: Int J Mol Sci. 2026 Nov 8;27(21):10009. doi: 10.3390/ijms272110009 (PMC13764317; doi:10.3390/ijms272110009)
Supplement: Supplementary file 1 [file ijms-27-10009-s001.zip › Figure S.pdf]

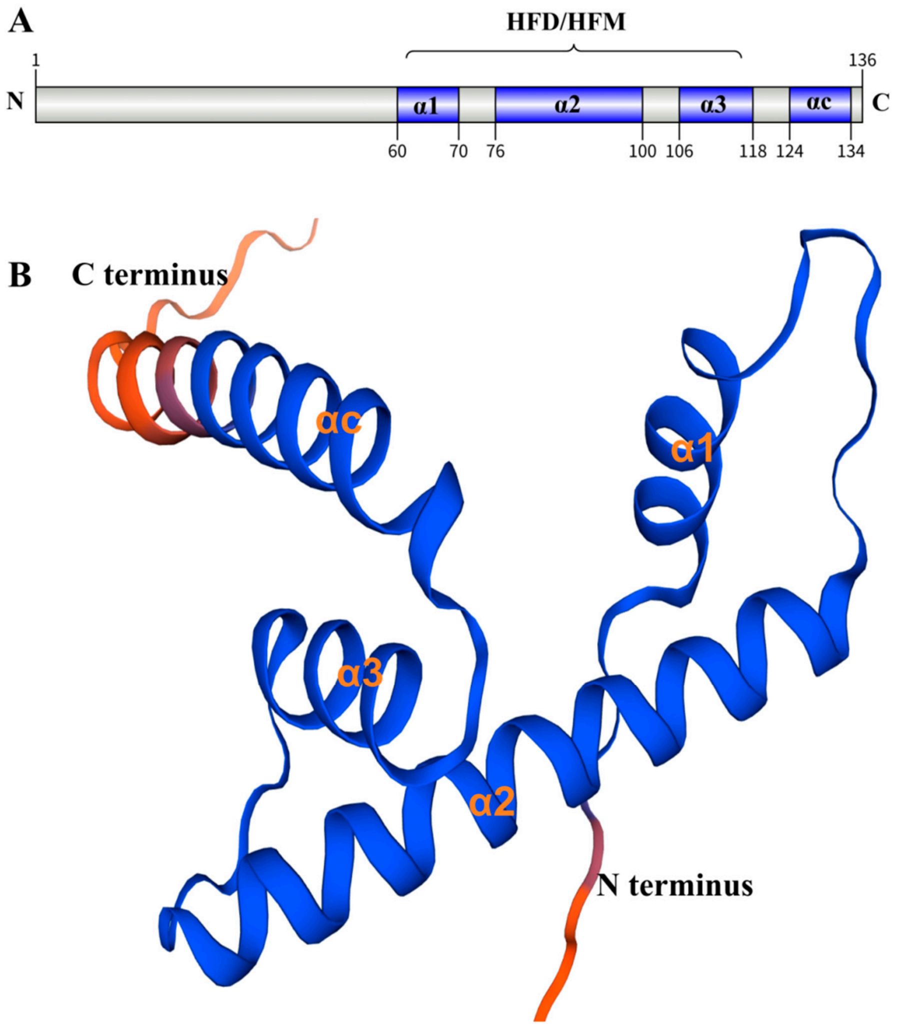

Figure S1. Predicted schematic diagram of domain architecture and three-dimensional conformation of wheat TaNF-YB2A protein. A Linear schematic representation of TaNF-YB2A protein architecture. The conserved histone-fold domain (HFD/HFM) contains the α-helices (α1, α2, α3 and αc). N-terminus and C-terminus are labelled. B Predicted 3D ribbon structure generated by Swiss-Model. Conserved domain is shown in blue; flexible N-terminal and C-terminal segments are in orange-yellow.

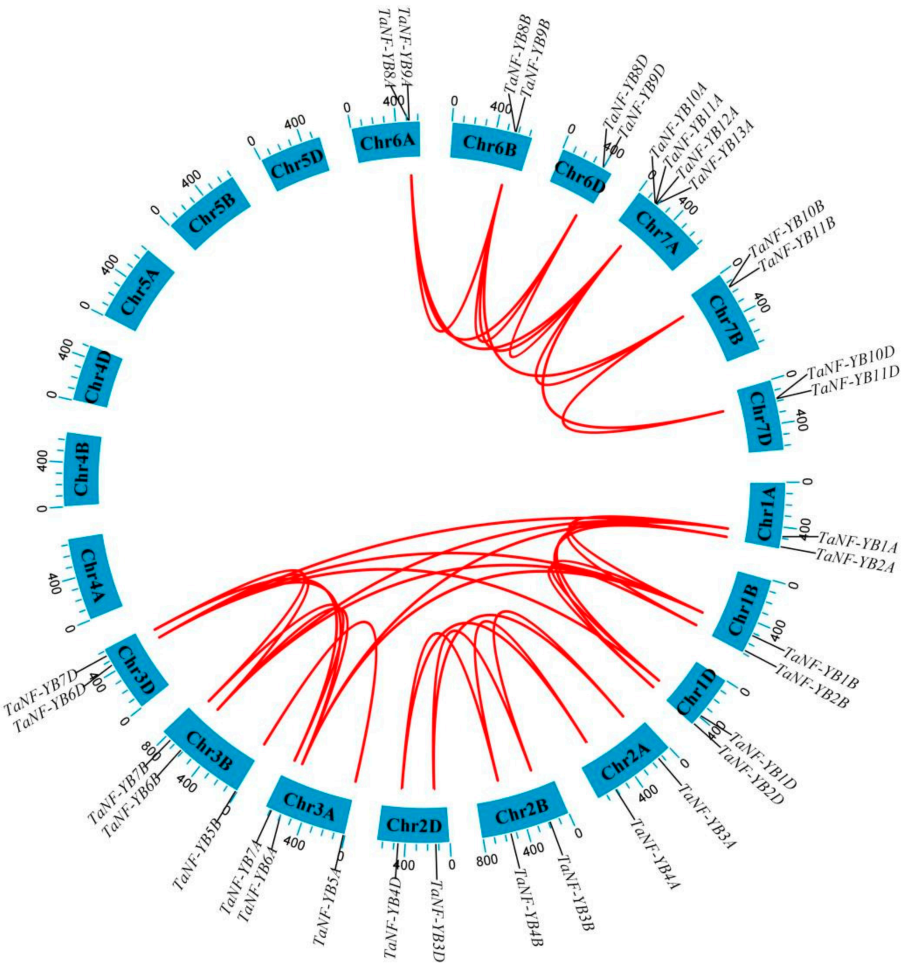

Figure S2. Predicted collinearity analysis of all *TaNF-YBs* in wheat. *TaNF-YBs* anchored to corresponding positions on wheat chromosomes, were analyzed for their duplications.
